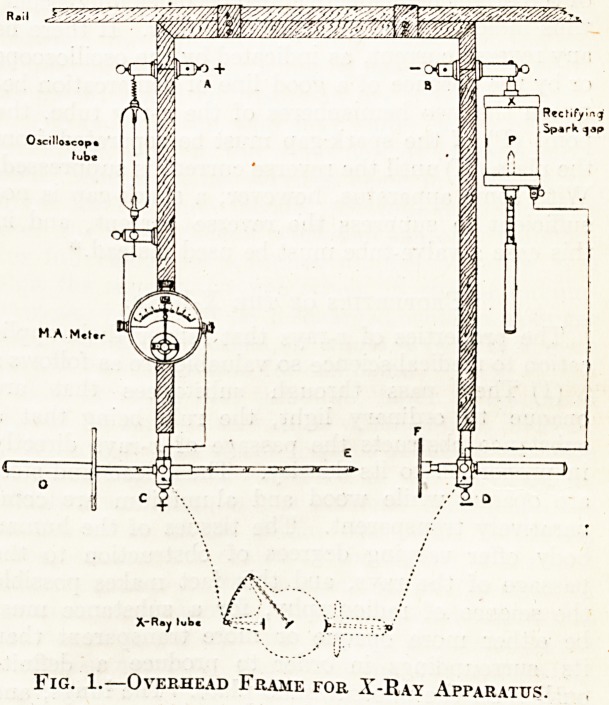# Fluoroscopy and Radiography

**Published:** 1912-11-09

**Authors:** Alfred C. Norman

**Affiliations:** House Surgeon at Durham County Eye Infirmary.


### ELECTRICITY IN MODERN MEDICINE.*

### XXI.—Fluoroscopy and Radiography.

By ALFRED C. NORMAN, M.D. Edin., House Surgeon at Durham County Eye Infirmary.

The following six paragraphs should be read in conjunction with Section XIX.

Fig. 1 shows diagrammatically the overhead frame which supports the various instruments belonging to the secondary circuit, and the arrangement of terminals and wires which connect them together in series. It will be noticed that the spintermeter (belonging to the induction coil) has been attached to the frame in such a way that the metal plate (F) is a fixture on the right arm, while the pointed rod (E), supported by the left arm of the frame, can be pushed towards or withdrawn from the plate (F) at will.

To complete the secondary circuit it is only necessary to connect the terminals, A and B, with the discharging pillars of the induction coil, and to connect the terminals, C and D, with the *x*-ray tube. With the apparatus arranged as shown in the diagram it is, however, absolutely essential that the terminal (A) be connected with the *positive pillar* (anode) and the terminal (B) with the *negative pillar* (cathode) of the coil, otherwise the rectifying spark-gap would not only *not* suppress the *reverse* current, but would actually obstruct the flow of *direct* current. The terminal (C) will, of course, always be *positive*, and the terminal (D) *negative*, and it will soon become a matter of habit with the radiographer to connect up his *x*-ray tube in the proper way—namely, the anode (or anti-cathode) of the tube with C, and the cathode (concave electrode) of the tube with D.

Let us now trace the course of the secondary circuit. Current is brought from the positive pillar of the induction coil to the terminal (A); from there it passes through the oscilloscope tube, only the lower wire of which becomes surrounded by a violet fluorescence if there be no reverse current; from the oscilloscope tube it passes through the milliamperemeter, deflecting the needle towards the right, and from there to the terminal (C). From this point it is conveyed by means of a connecting wire to the *x*-ray tube, and, having passed through the latter, it is carried by another wire to the terminal (D); from there it passes to the point (P) of the spark-gap, jumps across a little air-gap to the plate (X) of the spark-gap, travels to the terminal (B), and is thence conveyed to the negative pillar of the induction coil.

*The connecting cords*, which carry current to the *x*-ray tube, require a little consideration. The current in this circuit is never greater than a small fraction of an ampere, hence very fine wire can be employed to carry it. The writer uses No. 22 copper wire covered with a thin layer of gutta-percha; the latter gives to the wire a certain amount of rigidity, and the wire itself is light and easily handled. Another kind of wire often employed is the fine, silk-covered, tinsel wire originally made for connecting cords in galvanic treatment. For *x*-ray work these wires can be obtained conveniently mounted on spring reels (like little tape measures), which keep the wires always taut and so diminish the risk of their accidentally touching the patient.

The gutta-percha or silk covering these wires is not, of course, a sufficient insulation against the enormous voltage of the current they carry, hence they must not be touched while the coil is working, and, above all, they must not be allowed to approach the bulbous portion of the *x*-ray tube, otherwise a spark might easily puncture the latter.

Fig. 1.—Overhead Frame for X-Ray Apparatus.

Connecting cords can now be obtained so heavily insulated that, when new, they can be safely held in the hand while the coil is working. They are not to be recommended, however, for they are thick and heavy and difficult to manipulate, and, moreover, they give one a false sense of security, for their insulation is certain to break down sooner or later under the heavy voltage.

### Working the X-Ray Apparatus.

The apparatus, connected as described above, is always ready to furnish *x*-rays at a moment's notice; the necessary manipulations are as follows: After making sure that both switches on the switchboard are open, and that both rheostats are at "weak," we proceed to close the double-pole main switch in order to turn current on to the switchboard. Next we turn on the "motor" switch in order to set

* Previous articles appeared on Nov. 11, 25, Dec. 9, 30, Jan. 13, 27, Feb. 17, March 9, 30, April 20, May 4, 25, June 8, July 6, Aug. 3, 17, 31, Sept. 28, and Oct. 12, 26.

the interrupter revolving, and then we gradually increase the speed of the latter by means of the sliding contact. Our next procedure is to close the "coil" switch, which allows current to pass through the primary winding of the induction coil, and to gradually increase this current, by means of the crank rheostat, until the milliampere-meter indicates that the current flowing in the secondary circuit is approximately the "normal" current of the tube (see page 458 of The Hospital for August 3). Lastly, by means of the small sliding rheostat, we increase or diminish the speed of the interrupter until the output from the coil is at its maximum efficiency (see page 201 of The Hospital for May 25).

The $x$-ray tube should now be fluorescing brightly in one hemisphere, while the other hemisphere remains dark, and if a fluoroscope be held in front of the bright hemisphere it also becomes fluorescent, thus indicating the presence of $x$-rays. If there be any reverse current, as indicated by the oscilloscope or by the absence of a good line of demarcation between the two hemispheres of the $x$-ray tube, the point (P) of the spark-gap must be separated from the plate (X) until the reverse current is suppressed. With some apparatus, however, a spark-gap is not sufficient to suppress the reverse current, and in this case a valve-tube must be used instead.*

### Properties of the X-Rays.

The properties of $x$-rays that render their application to medical science so valuable are as follows:

(1) They pass through substances that are opaque to ordinary light, the rule being that a substance obstructs the passage of $x$-rays directly in proportion to its density. Thus, lead and steel are opaque while wood and aluminium are comparatively transparent. The tissues of the human body offer varying degrees of obstruction to the passage of the rays, and this fact makes possible the science of radiography, for a substance must be either more opaque or more transparent than its surroundings in order to produce a definite outline on the photographic plate. The lungs, and the intestines when filled with air, are the most transparent tissues of the human body, the skin, muscles, and solid viscera are less so, while the bones and teeth are least transparent of all, because of the density of the calcium salts which they contain. For the same reason a renal or vesical calculus or a calcified gland is more opaque than its surroundings, and so produces a good radiographic shadow.

(2) The $x$-rays produce a chemical effect upon a photographic plate similar to that of ordinary light; hence we are able to obtain permanent records—radiographs—of the $x$-ray shadows cast by the various tissues of the body.

(3) They excite fluorescence in certain chemicals, the amount of fluorescence being directly proportional to the quantity of rays that reach the chemical. This property is utilised in fluoroscopy.

(4) $X$-rays given off by different tubes have wave-lengths varying with the degree of exhaustion of the tube; and the penetrating power of the rays varies with their wave length. For practical purposes the penetrating power of the different rays may be classified into three degrees—"hard," "medium," and "soft," and in radiography much depends upon the selection of a tube that gives out rays of a penetration suitable for the work in hand.

(5) $X$-rays have an action upon the tissues of the body that may be either pathological or therapeutic—this depending upon the quantity and penetrating power of the rays applied.

### Fluoroscopy.

Fluoroscopy consists in examining on the fluorescent screen the shadows of varying density cast by the tissues of the human body. If the $x$-rays were visible to the eye no screen would be required, but, as they are not, we have to convert their vibrations into a form of visible light (fluorescence) in order to perceive them. The examination must be made in a darkened room, and the operator should be in the dark for at least ten minutes beforehand in order to adapt his retinæ to the deficient illumination.

Suppose we wish to locate a needle embedded in the flesh of a patient's hand. The hand is pressed against the back of the screen and the latter is held in the path of the $x$-rays. Except where it is covered by the hand the whole screen fluoresces brightly. The needle, being most opaque, obstructs the passage of nearly all the rays that fall upon it, hence it throws an intensely black shadow upon the screen. The bones allow more rays to pass than does the needle, consequently they throw a less dense shadow on the screen. The flesh hardly obstructs the rays at all, therefore its shadow is hardly perceptible, or, in other words, the part of the screen in front of the flesh is fluorescing almost as brightly as the part on which the rays strike directly. Thus we see that the fluoroscopic image is really more than a shadow—it is a temporary chart of the densities of the various tissues through which the $x$-rays pass.

The above description applies only when a tube of medium penetrating power is used. If we employ a very soft tube the flesh will obstruct almost all the $x$-rays, and the image on the screen will be a dense black shadow presenting a great contrast with the surrounding screen, but with practically no differentiation between needle, flesh, and bones. To go to the opposite extreme, if a very hard tube be employed the bones will be almost as transparent as the flesh, consequently the shadow will be a thin grey one, and there will be very little contrast between flesh, bone, and the surrounding screen.

*(To be continued.)*

---

* The overhead wood frame can be obtained from Messrs. Schall & Son, 75 New Cavendish Street, W. With terminals, copper connecting wires, fittings for the spinter-meter, and a board to support the milliampere-meter it costs 33s.; or complete, with milliampere-meter, oscilloscope tube, and rectifying spark-gap, the price is £6 6s. 6d.

## Figures and Tables

**Fig. 1. f1:**